# PanViTa: Pan Virulence and resisTance analysis

**DOI:** 10.3389/fbinf.2023.1070406

**Published:** 2023-02-07

**Authors:** Diego Lucas Neres Rodrigues, Juan Carlos Ariute, Francielly Morais Rodrigues da Costa, Ana Maria Benko-Iseppon, Debmalya Barh, Vasco Azevedo, Flávia Aburjaile

**Affiliations:** ^1^ Preventive Veterinary Medicine Departament, Veterinary School, Universidade Federal de Minas Gerais, Belo Horizonte, Brazil; ^2^ Genetics Department, Universidade Federal de Pernambuco, Recife, Brazil; ^3^ Departament of Genetics, Ecology and Evolution, Universidade Federal de Minas Gerais, Belo Horizonte, Brazil; ^4^ Institute of Integrative Omics and Applied Biotechnology (IIOAB), Purba Medinipur, India

**Keywords:** bioinformatics tools, data visualization, multiomics analysis, pathogens, resistome

## 1 Introduction

Along with the steady increase of multi-resistant and extensively virulent microorganisms, the genomic approach has become an essential ally in the search for genetic factors related to microbial pathogenicity (Mbelle et al., 2019). Thus, the call for tools capable of handling a large scale of information in a short period of time has increased (Suárez-Díaz, 2010).

The fact is that genomic information tends to be difficult to interpret due to the high information density present in several datasets (Nusrat et al., 2019). Moreover, the visualization of genomic analysis results becomes more complex whenever new genomes are added to the initial dataset and additional information is computed.

PanViTa (Pan Virulence and resisTance Analysis) was developed with the concepts of scalability and agility in mind. It is a tool made entirely in Python3 (Van Rossum and Drake, 2009), focusing on the analysis of multi-omic bacterial data based on complete or draft genomes. This tool was initially designed to handle data annotated by the PROKKA pipeline (Seemann, 2014) using GenBank files as input (.gbk or.gbff). However, it has been adapted to receive any GenBank file—with some reservations.

The tool is available on GitHub through the link https://github.com/dlnrodrigues/panvita.

## 2 Materials and methods

### 2.1 Implementation

The tool uses databases to obtain biological information available through the web, including CARD (Comprehensive Antimicrobial Resistance Database) (Alcock et al., 2020) and BacMet2 (Antibacterial Biocide and Metal Resistance Genes Database) (Pal et al., 2014) for resistance analysis, and VFDB (Virulence Factor Database) (Liu et al., 2019) for virulence analysis. The user can select any of the databases initially *via* the command line. BLASTp (Altschul et al., 1990; Mount, 2007) was selected in conjunction with the DIAMOND algorithm to compare the user data with the database reference (Buchfink et al., 2015).

For some features of the developed tool, it was necessary to take advantage of some existing libraries and modules in the native language. Therefore, the use of the Python3 version is recommended. Besides intrinsic modules and libraries (sys, OS, shutil, and math), the program also needs to import other modules and libraries: wget, to get external data and update databases and dependencies whenever necessary; pandas (Reback et al., 2021) for matrix construction and manipulation; seaborn (Waskom et al., 2020) and matplotlib (Hunter, 2007) both for the final plotting of graphical results. PanViTa requires 17 Mb of hard disk space for installation.

To obtain the final result, the program performs the following steps: (I) extracts the amino acid sequences of the predicted proteome from each GenBank file; (II) extracts the positions of the coding sequences of all proteins in the genome from each GenBank file; (III) aligns the predicted proteome with the selected database using DIAMOND-BLASTp; (IV) filters the results in the tabular output file that match the identity and coverage parameters (by default, results above 70% identity and 70% coverage are considered); (V) summarizes the results in a similarity-based matrix: X represents the genes with a match higher than the defined cutoff and Y represents the strains given as input; (VI) uses the summarized results to generate a clustermap plot based on Euclidean distance to determine data clusters by proximity; (VII) plots the development of each subpartition in core- and pan-; (VIII) checks and summarizes the gene results by specific strain. The flow chart containing the software steps is shown in Supplementary Figure S1.

**FIGURE 1:**
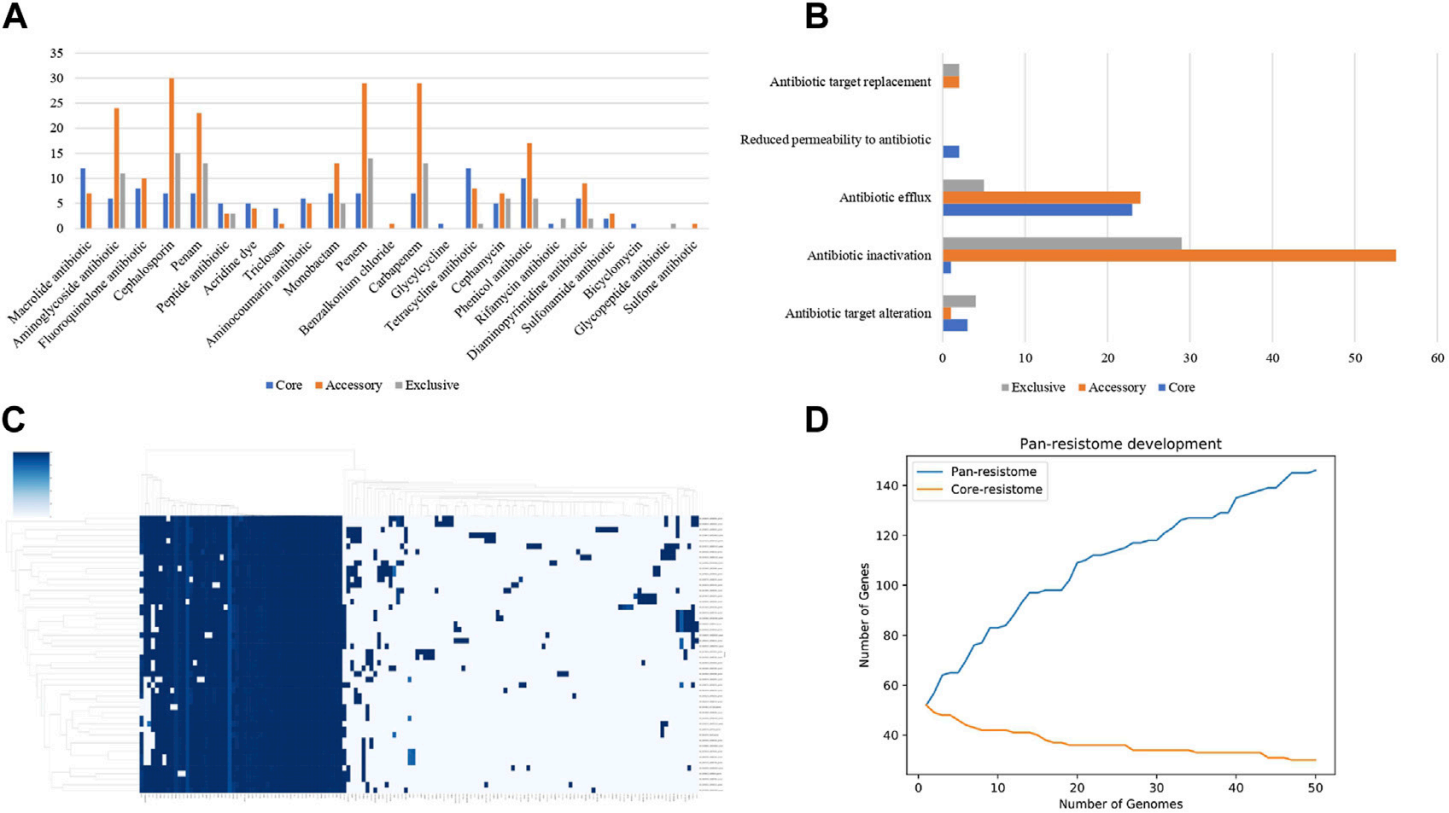
**(A)** graphical representation of the distribution of data referring to the number of resistance genes found by type of antimicrobials; **(B)** a visual representation of the data distribution regarding the number of resistance genes found by a variety of related resistance mechanisms; **(C)** representative clustermap based on the relationship of presence and absence of each gene found in the pan-resistome; **(D)** graph of genomic development based on the predicted resistome. In blue, the pan-resistome development curve is represented, and in orange, the core-resistome development curve.

Figure 1 represents some of the outputs generated using the PanViTa tool.

### 2.2 Comparative analysis

The performance of PanViTa was compared with other tools developed with a similar purpose: Abricate (Seemann, 2020) and ResFinder (Bortolaia et al., 2020). For comparison, genomes from different *Acinetobacter baumannii*, *Escherichia coli*, and *Pseudomonas aeruginosa* were selected. Table 1 represents the results obtained. The assembly codes from the strains of *A. baumannii*, *E. coli*, and *P. aeruginosa* are presented on Supplementary Table S1.

**TABLE 1:** Results obtained based on the comparison of the three tools analyzed.

		*Acinetobacter baumannii*	*Escherichia coli*	*Pseudomonas aeruginosa*
Number of genomes		100	65	50
Genome size (Mb) ≅		4.03	5.24	7.15
Time	PanViTa	00:02:08	00:01:36	00:01:23
	Abricate	00:04:34	00:04:29	00:08:59
	ResFinder	01:13:52	01:06:26	01:06:13
Number of genes	PanViTa	145	112	146
	Abricate	119	120	119
	ResFinder	99	65	91

For standardization purposes only the CARD database was added to the comparison analysis with Abricate.

The analyses were made on a desktop computer with OS Linux-Ubuntu viewing 8 GB of RAM and four cores (Intel^®^ Core i5-3570 CPU 3.40 GHz).

## 3 Comparative results

Regarding the time spent, PanViTa was superior to the other tools compared. This fact can be related to both DIAMOND and BLASTp, which increase the alignment speed. The analysis of resistance genes is relative and dependent on the database, not being subject to direct comparison. Another important fact is the difference between the alignment matrices since PanViTa uses the amino acid sequences as the primary input, and Abricate and ResFinder consider nucleotide sequences. Besides, only PanViTa generates visual output.

The specific results obtained for each species are available in Supplementary Table S2.

## 4 Outputs

PanViTa provides some results based on the presence/absence of genes. Through this methodology it is possible to swiftly extract quantitative information about the action mechanisms of certain gene products, as well as which compounds are related to them.

### 4.1 Presence/absence matrix

One of the main outputs is a presence/absence matrix for each database, containings all identity values for each gene in each strain. All values are retrieved from multiple alignments against the previous selected database. Only the highest identity values per gene are considered for matrix building.

### 4.2 Clustermap

Euclidean distance is used as the metric to plot the clustermaps. In this way, it is possible to identify which genes are statistically related to each other. In addition, this data also enables to infer which strains are more or less related to each other using only a few resistance or pathogenicity genes presence.

### 4.3 Strain-specific genes

These both outputs are related to presence/absence statistics. With the usage of these outputs, it is possible to obtain the number and families of genes found on each bacterial strain properly, as well as the number of strains that share the same gene.

### 4.4 Virulence and resistance factors

PanViTa generates a single file for each strain containg the positions of CDSs related to specific virulence and resistance factors found on previous analysis. This file keeps the information from the original. gbk file, otherwise, if the original genome is not complete and has multiple contigs, the positions will be consecutive and addictive. In other words, if there’s more than one contig on GenBank’s file, to the positions extracted from consecutives contigs will be added the value of the length of the previous contigs.

### 4.5 Pan-ome curve

The pan-omic curve is an approximation of the pan-genome curve obtained from a basic pangenomic analysis. It is important to note, however, that this output has small statistical power because it is a plot of gene distribution from both sections of the pan-genomic approach—core genome and accessory genome. Non-etheless, it is interesting to observe, for example, if the pan-ome curve reached a stable point. Otherwise, the dataset considered for the analysis has a chance to continue to get over new resistance or virulence factors.

### 4.6 Antibiotics

When the selected database is CARD or BacMet2, it is possible to obtain a table that quantifies genes related to each antibiotic class obtained, being grouped by sub partition along the pan-resistome (central, accessory and exclusive). In this way, it is possible to assess the presence of certain target factors in more specific portions of the sample.

### 4.7 Other functions

PanViTa also has a genome acquisition module that allows the download of genomes available on the NCBI platform. For this, it is only necessary to use the. csv file generated during the genome search as input. In addition, it is possible to automatically generate the script for annotation using the prokka pipeline, and obtain the host and related disease metadata using the available biosample number.

## Data Availability

The original contributions presented in the study are included in the article/Supplementary Material, further inquiries can be directed to the corresponding author.
