# Supplementary material for: PanViTa: Pan Virulence and resisTance analysis
Source: Front Bioinform. 2023 Feb 7;3:1070406. doi: 10.3389/fbinf.2023.1070406 (PMC9942593; doi:10.3389/fbinf.2023.1070406)
Supplement: Supplementary file 1 [file DataSheet2.pdf]

Diego Lucas Neres Rodrigues[1], Juan Carlos Ariute[1,2], Francielly Morais Rodrigues da Costa[3], Ana Maria Benko-Iseppon[2], Debmalya Barh[3,4], Vasco Azevedo[3†] and Flávia Aburjaile[1*†]

[1]Preventive Veterinary Medicine Departament, Veterinary School, Universidade Federal de Minas Gerais, Belo Horizonte 31270-901, Brazil

[2]Genetics Department, Universidade Federal de Pernambuco, Recife 50740-600, Brazil

[3]Department of Genetics, Ecology and Evolution, Institute of Biological Sciences, Federal University of Minas Gerais, Belo Horizonte 31270-901, Brazil

[4]Institute of Integrative Omics and Applied Biotechnology (IIOAB), Nonakuri, Purba Medinipur 721172, India.[†]These authors contributed equally to this work.

***Correspondence:**
Flávia Aburjaile
faburjaile@gmail.com

Supplementary Material

1 Supplementary Figure

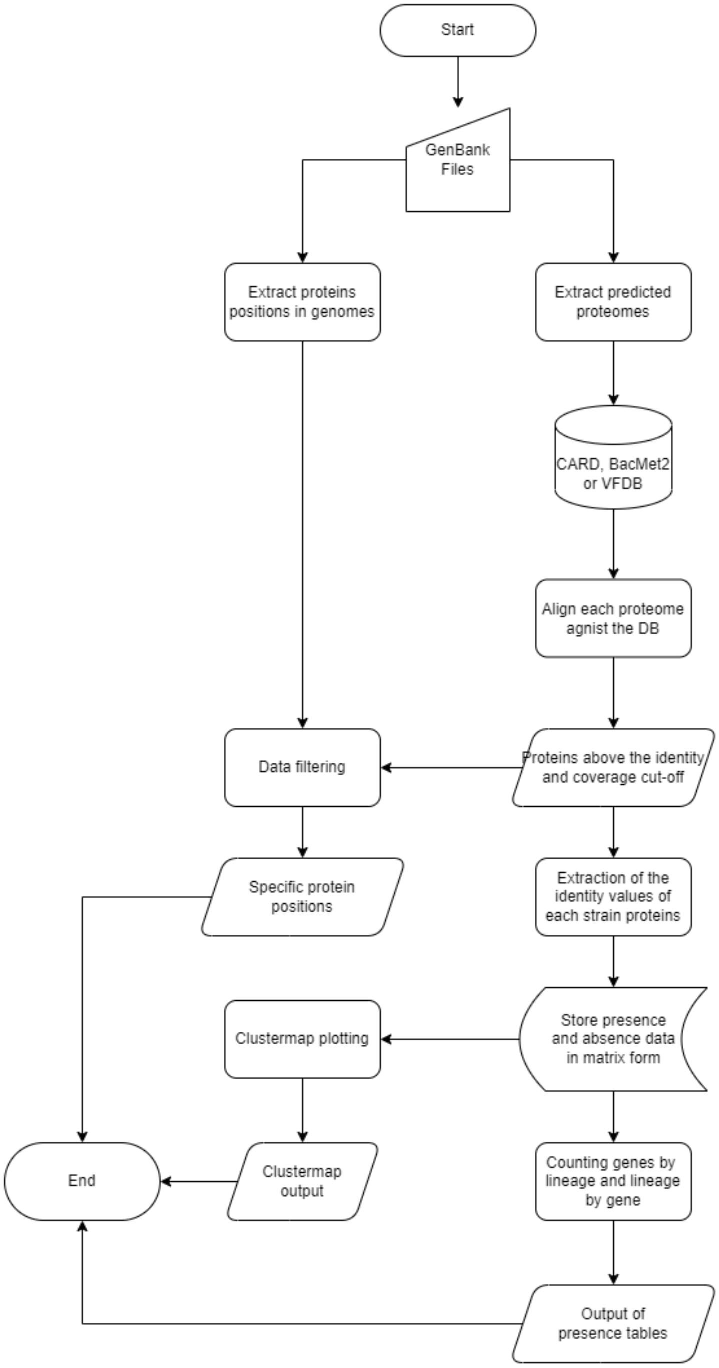

Supplementary Figure S1. PanViTa pipeline overview that represents the flow of steps necessary for developing the activity proposed by the tool.

2 Supplementary Tables

Supplementary Table S1. Summary of public genomes used in tool performance analysis. The table shows the access data for the genomes used for the comparative performance analysis of the PanViTa tool.

Species	Strain	Assembly	Size(Mb)
Acinetobacter baumannii	3207	GCA_001636235.1	4.0920
Acinetobacter baumannii	5845	GCA_003522885.1	3.9900
Acinetobacter baumannii	6200	GCA_000814345.1	4.0740
Acinetobacter baumannii	6507	GCA_009455505.1	4.0921
Acinetobacter baumannii	7804	GCA_003431385.1	4.3420
Acinetobacter baumannii	7835	GCA_009497995.1	4.0958
Acinetobacter baumannii	7847	GCA_003516005.1	4.0806
Acinetobacter baumannii	9102	GCA_003522845.1	4.0668
Acinetobacter baumannii	9201	GCA_003522665.1	4.1282
Acinetobacter baumannii	10042	GCA_003522785.1	4.1519
Acinetobacter baumannii	10324	GCA_003522705.1	3.8624
Acinetobacter baumannii	09A16CRGN0014	GCA_003931755.1	3.9433
Acinetobacter baumannii	09A16CRGN003B	GCA_003931775.1	3.9431
Acinetobacter baumannii	11A1213CRGN008	GCA_004101745.1	3.9449
Acinetobacter baumannii	11A1213CRGN055	GCA_004101725.1	3.9341
Acinetobacter baumannii	11A1213CRGN064	GCA_008244865.1	3.9353
Acinetobacter baumannii	11A1314CRGN088	GCA_008244905.1	3.9351
Acinetobacter baumannii	11A1314CRGN088	GCA_004101685.1	3.9375
Acinetobacter baumannii	11A14CRGN003	GCA_004101705.1	3.9407
Acinetobacter baumannii	11W359501	GCA_006385075.1	4.1302
Acinetobacter baumannii	15A34	GCA_002082685.1	3.9387

Supplementary Material

Acinetobacter baumannii	15A5	GCA_002082705.1	4.0493
Acinetobacter baumannii	1656-2	GCA_000188215.1	4.0231
Acinetobacter baumannii	2008S11-069	GCA_003359255.2	4.1101
Acinetobacter baumannii	29FS20	GCA_013394285.1	4.1333
Acinetobacter baumannii	31FS3-2	GCA_013394265.1	3.9312
Acinetobacter baumannii	810CP	GCA_003345235.1	4.1111
Acinetobacter baumannii	A1	GCA_000830055.1	3.9177
Acinetobacter baumannii	A1296	GCA_002504145.1	3.8107
Acinetobacter baumannii	A1429	GCA_013377175.1	4.1619
Acinetobacter baumannii	A320_RUH134	GCA_007221455.1	3.8965
Acinetobacter baumannii	A388	GCA_002741415.1	4.0321
Acinetobacter baumannii	A52	GCA_004028375.1	4.0233
Acinetobacter baumannii	A85	GCA_002210065.1	4.1446
Acinetobacter baumannii	AB0057	GCA_000021245.2	4.0639
Acinetobacter baumannii	AB030	GCA_000746645.1	4.3358
Acinetobacter baumannii	AB031	GCA_000746605.1	3.8033
Acinetobacter baumannii	AB042	GCA_001941765.1	4.0047
Acinetobacter baumannii	AB043	GCA_008630895.1	3.9617
Acinetobacter baumannii	Ab04-mff	GCA_001077655.1	4.1923
Acinetobacter baumannii	AB307-0294	GCA_002803025.2	3.7595
Acinetobacter baumannii	AB34299	GCA_002009115.1	3.9830
Acinetobacter baumannii	Ab4568	GCA_002762115.1	4.0005
Acinetobacter baumannii	Ab4653	GCA_002762155.1	3.9139
Acinetobacter baumannii	Ab4977	GCA_002762095.1	3.9669
Acinetobacter baumannii	AB5075-UW	GCA_000963815.1	4.0670
Acinetobacter	ab736	GCA_002116925.1	3.9904

baumannii			
Acinetobacter baumannii	Aba	GCA_003288775.1	3.9205
Acinetobacter baumannii	Ab-B004d-c	GCA_012931645.1	4.1591
Acinetobacter baumannii	Ab-C102	GCA_012931605.1	3.9401
Acinetobacter baumannii	Ab-C63	GCA_012931665.1	3.9659
Acinetobacter baumannii	AbCAN2	GCA_009833005.1	3.8098
Acinetobacter baumannii	ABCR01	GCA_009498335.1	4.0304
Acinetobacter baumannii	Ab-D10a-a	GCA_012931625.1	4.1747
Acinetobacter baumannii	ABF9692	GCA_012974585.1	4.1268
Acinetobacter baumannii	AbH12O-A2	GCA_000761175.1	3.8758
Acinetobacter baumannii	ABNIH28	GCA_002902885.1	4.0788
Acinetobacter baumannii	AbPK1	GCA_002753915.1	4.1473
Acinetobacter baumannii	ABUH763	GCA_001674475.2	4.0145
Acinetobacter baumannii	ABUH773	GCA_001668465.2	3.8857
Acinetobacter baumannii	ABUH793	GCA_001669145.2	4.1079
Acinetobacter baumannii	ABUH796	GCA_001674505.2	3.9438
Acinetobacter baumannii	AC29	GCA_000695855.3	3.9351
Acinetobacter baumannii	AC30	GCA_000307975.2	3.9229
Acinetobacter baumannii	ACICU	GCA_005519135.1	4.0136
Acinetobacter baumannii	ACN21	GCA_004768705.1	4.0456
Acinetobacter baumannii	AF-401	GCA_001896005.1	4.2676
Acinetobacter baumannii	AF-673	GCA_001895985.1	4.0931
Acinetobacter baumannii	AR_0052	GCA_003547115.1	4.2889
Acinetobacter baumannii	AR_0056	GCA_002947415.1	4.1884
Acinetobacter baumannii	AR_0063	GCA_002947845.1	4.1018
Acinetobacter baumannii	AR_0070	GCA_003431865.1	4.3173

Supplementary Material

Acinetobacter baumannii	AR_0078	GCA_002948925.1	3.8719
Acinetobacter baumannii	AR_0083	GCA_002996805.1	4.1582
Acinetobacter baumannii	AR_0088	GCA_003006035.1	4.2283
Acinetobacter baumannii	AR_0101	GCA_003010675.1	4.2499
Acinetobacter baumannii	AR_0102	GCA_003010655.1	4.0603
Acinetobacter baumannii	ATCC_17978	GCA_013372085.1	4.0301
Acinetobacter baumannii	ATCC_17978_substr._PMR-High	GCA_004797155.2	4.0163
Acinetobacter baumannii	ATCC_17978-mff	GCA_001077675.1	4.0067
Acinetobacter baumannii	ATCC_19606	GCA_009759685.1	3.9904
Acinetobacter baumannii	ATCC_BAA-1790	GCA_008033255.1	4.0303
Acinetobacter baumannii	AYE	GCA_000069245.1	4.0487
Acinetobacter baumannii	AYP-A2	GCA_002761575.1	4.1027
Acinetobacter baumannii	B8300	GCA_001077965.2	3.8501
Acinetobacter baumannii	B8342	GCA_001077555.2	3.9478
Acinetobacter baumannii	BAL062	GCA_900088705.1	4.0508
Acinetobacter baumannii	BJAB07104	GCA_000419385.1	4.0422
Acinetobacter baumannii	BJAB0715	GCA_000419405.1	4.0539
Acinetobacter baumannii	BJAB0868	GCA_000419425.1	4.0058
Acinetobacter baumannii	C25	GCA_008807055.1	4.0294
Acinetobacter baumannii	CA-17	GCA_001721705.1	3.7193
Acinetobacter baumannii	CAM180-1	GCA_008802935.1	3.9232
Acinetobacter baumannii	CBA7	GCA_002082645.1	4.1085
Acinetobacter baumannii	CIAT758	GCA_004758865.1	4.1724
Acinetobacter baumannii	CIP70.10	GCA_001457535.1	3.9363
Acinetobacter baumannii	CMC-CR-MDR-Ab4	GCA_001922205.1	4.1560
Acinetobacter	CMC-CR-MDR-Ab66	GCA_001922245.1	4.1742

baumannii			
Acinetobacter baumannii	CMC-MDR-Ab59	GCA_001922225.1	4.0764
Acinetobacter baumannii	CUVET-MIC596	GCA_012974845.1	4.0804
Pseudomonas aeruginosa	NCTC10332	GCA_001457615.1	6.32
Pseudomonas aeruginosa	PAC1	GCA_013201115.1	7.56
Pseudomonas aeruginosa	Carb01 63	GCA_000981825.1	7.5
Pseudomonas aeruginosa	Pa1207	GCA_002208645.1	7.41
Pseudomonas aeruginosa	RIVM-EMC2982	GCA_002085605.1	7.38
Pseudomonas aeruginosa	1811-13R031	GCA_009676765.1	7.34
Pseudomonas aeruginosa	1811-18R001	GCA_009676785.1	7.34
Pseudomonas aeruginosa	F30658	GCA_001516265.1	7.27
Pseudomonas aeruginosa	AR442	GCA_003073795.1	7.27
Pseudomonas aeruginosa	Pa58	GCA_002192495.1	7.24
Pseudomonas aeruginosa	AR_0353	GCA_002968655.1	7.28
Pseudomonas aeruginosa	1	GCA_900497025.1	7.23
Pseudomonas aeruginosa	AG1	GCA_009662315.1	7.19
Pseudomonas aeruginosa	YTSEY8	GCA_016745115.1	7.18
Pseudomonas aeruginosa	AR_0357	GCA_002968955.1	7.16
Pseudomonas aeruginosa	SE5458	GCA_013394475.2	7.16
Pseudomonas aeruginosa	TJ2019-022	GCA_016105505.1	7.16
Pseudomonas aeruginosa	SE5369	GCA_013393685.1	7.15
Pseudomonas aeruginosa	Pa127	GCA_002205355.1	7.15
Pseudomonas aeruginosa	AR439	GCA_003073895.1	7.58
Pseudomonas aeruginosa	AR445	GCA_003073735.1	7.13
Pseudomonas aeruginosa	FDAARGOS_570	GCA_003813025.1	7.16
Pseudomonas aeruginosa	Y82	GCA_003369755.1	7.11

Pseudomonas aeruginosa	SE5429	GCA_016745175.1	7.1
Pseudomonas aeruginosa	24Pae112	GCA_003433235.1	7.1
Pseudomonas aeruginosa	401853	GCA_014155905.1	7.09
Pseudomonas aeruginosa	NCGM257	GCA_001547955.1	7.09
Pseudomonas aeruginosa	A-I-1	GCA_015697665.1	7.09
Pseudomonas aeruginosa	H26027	GCA_003798105.1	7.08
Pseudomonas aeruginosa	SE5331	GCA_013393665.2	7.06
Pseudomonas aeruginosa	MRSN12280	GCA_003028335.1	7.05
Pseudomonas aeruginosa	Pa1242	GCA_002205375.1	7.05
Pseudomonas aeruginosa	RW109	GCA_900243355.1	7.76
Pseudomonas aeruginosa	IMP-13	GCA_003950015.1	7.18
Pseudomonas aeruginosa	CMC-097	GCA_016064595.1	7.04
Pseudomonas aeruginosa	SE5357	GCA_016745155.1	7.04
Pseudomonas aeruginosa	E6130952	GCA_002085755.1	7.08
Pseudomonas aeruginosa	K34-7	GCA_003206535.1	7.04
Pseudomonas aeruginosa	268	GCA_003641125.1	7.03
Pseudomonas aeruginosa	FDAARGOS_505	GCA_003813005.1	7.03
Pseudomonas aeruginosa	BAMCPA07-48	GCA_001632245.1	7.02
Pseudomonas aeruginosa	PA7790	GCA_001870265.1	7.07
Pseudomonas aeruginosa	P33	GCA_015832055.1	7.12
Pseudomonas aeruginosa	AR_0230	GCA_002968695.1	7.09
Pseudomonas aeruginosa	CCUG 51971	GCA_008195485.1	7.01
Pseudomonas aeruginosa	Pa124	GCA_002192475.1	7.01
Pseudomonas aeruginosa	PA11803	GCA_001792875.1	7.01
Pseudomonas aeruginosa	SP4527	GCA_003991465.1	7.01
Pseudomonas	FDAARGOS_571	GCA_003812885.1	7

aeruginosa			
Pseudomonas aeruginosa	PASGNDM699	GCA_002104595.1	6.99
Escherichia coli	RM10386	GCA_003112225.1	5.96779
Escherichia coli	FRIK944	GCA_001695515.1	5.8662
Escherichia coli	E2865	GCA_003966465.1	6.00792
Escherichia coli	FRIK2455	GCA_001651965.2	5.77552
Escherichia coli	FRIK2069	GCA_001651925.2	5.74083
Escherichia coli	RM8385	GCA_003112165.1	5.73281
Escherichia coli	JEONG-1266	GCA_001558995.2	5.57459
Escherichia coli	E2855	GCA_003966425.1	5.93508
Escherichia coli	C11	GCA_001900355.1	5.41457
Escherichia coli	RM9872	GCA_003586065.1	5.47544
Escherichia coli	E2863	GCA_003966445.1	5.75166
Escherichia coli	C3	GCA_001900515.1	5.41327
Escherichia coli	C5	GCA_001900535.1	5.63396
Escherichia coli	C7	GCA_001901425.1	5.37241
Escherichia coli	RM9131	GCA_003288295.1	5.43269
Escherichia coli	RM9975	GCA_003288275.1	5.43242
Escherichia coli	1517k	GCA_004377995.2	5.16434
Escherichia coli	64.1	GCA_014170675.1	5.487
Escherichia coli	144	GCA_002310595.1	5.36125
Escherichia coli	CFS3292	GCA_009867015.2	5.55817
Escherichia coli	CFS3273	GCA_009866945.1	5.53688
Escherichia coli	MRY15-117	GCA_002357895.1	5.29507
Escherichia coli	730V1	GCA_014771275.1	5.218
Escherichia coli	181.1	GCA_014170615.1	5.5179
Escherichia coli	746	GCA_002310655.1	5.31928
Escherichia coli	1105	GCA_002310635.1	5.13375
Escherichia coli	RM10042	GCA_003112205.1	5.26171
Escherichia coli	RM10466	GCA_003176855.1	5.27288
Escherichia coli	MRY15-131	GCA_002357875.1	5.21995
Escherichia coli	317	GCA_002310575.1	5.13591
Escherichia coli	C10	GCA_001900335.1	5.02169
Escherichia coli	2014-01-7375	GCA_904711265.1	5.27047
Escherichia coli	C4	GCA_001900315.1	4.99048
Escherichia coli	1428	GCA_002310695.1	5.30974
Escherichia coli	1303	GCA_000829985.1	5.15693
Escherichia coli	CFS3313	GCA_009867035.1	5.23538
Escherichia coli	PSUO2	GCA_002215095.1	5.03526
Escherichia coli	C8	GCA_001900555.1	5.09612
Escherichia coli	1190	GCA_002310615.1	4.98704
Escherichia coli	268.2	GCA_014170975.1	5.07345

Escherichia coli	3R	GCA_011067085.1	5.27296
Escherichia coli	ECOL-18-VL-LA-PA-Ryan-0026	GCA_007012305.1	5.13531
Escherichia coli	AMSCJX04	GCA_013389615.1	5.31111
Escherichia coli	C1	GCA_001900295.1	4.84302
Escherichia coli	PT109	GCA_009761715.1	4.99434
Escherichia coli	C2	GCA_001900495.1	4.81824
Escherichia coli	ECC-1470	GCA_000831565.1	4.90381
Escherichia coli	CFS3246	GCA_009866925.1	4.99515
Escherichia coli	873.1	GCA_014770895.1	5.2934
Escherichia coli	97.3	GCA_014771055.1	5.1357
Escherichia coli	SC516	GCA_002812545.1	4.7924
Escherichia coli	1943	GCA_002310555.1	5.00072
Escherichia coli	1223	GCA_002310715.1	4.95157
Escherichia coli	127	GCA_002310675.1	5.09128
Escherichia coli	1283	GCA_002310735.1	4.92005
Escherichia coli	54	GCA_012278675.1	4.77038
Escherichia coli	AMSCJX03	GCA_013391825.1	4.81981
Escherichia coli	YY76-1	GCA_008080655.1	4.81566
Escherichia coli	C9	GCA_001900575.1	4.67746
Escherichia coli	276.2	GCA_014170595.1	5.32583
Escherichia coli	56.2	GCA_014170695.1	5.30872
Escherichia coli	PSUO78	GCA_002215115.1	5.23057
Escherichia coli	34.1	GCA_014170715.1	4.97826
Escherichia coli	162.2	GCA_014170735.1	4.75087
Escherichia coli	101.3	GCA_014170945.1	4.86155
