# Supplementary figures and images for: PanViTa: Pan Virulence and resisTance analysis
Source: Front Bioinform. 2023 Feb 7;3:1070406. doi: 10.3389/fbinf.2023.1070406 (PMC9942593; doi:10.3389/fbinf.2023.1070406)

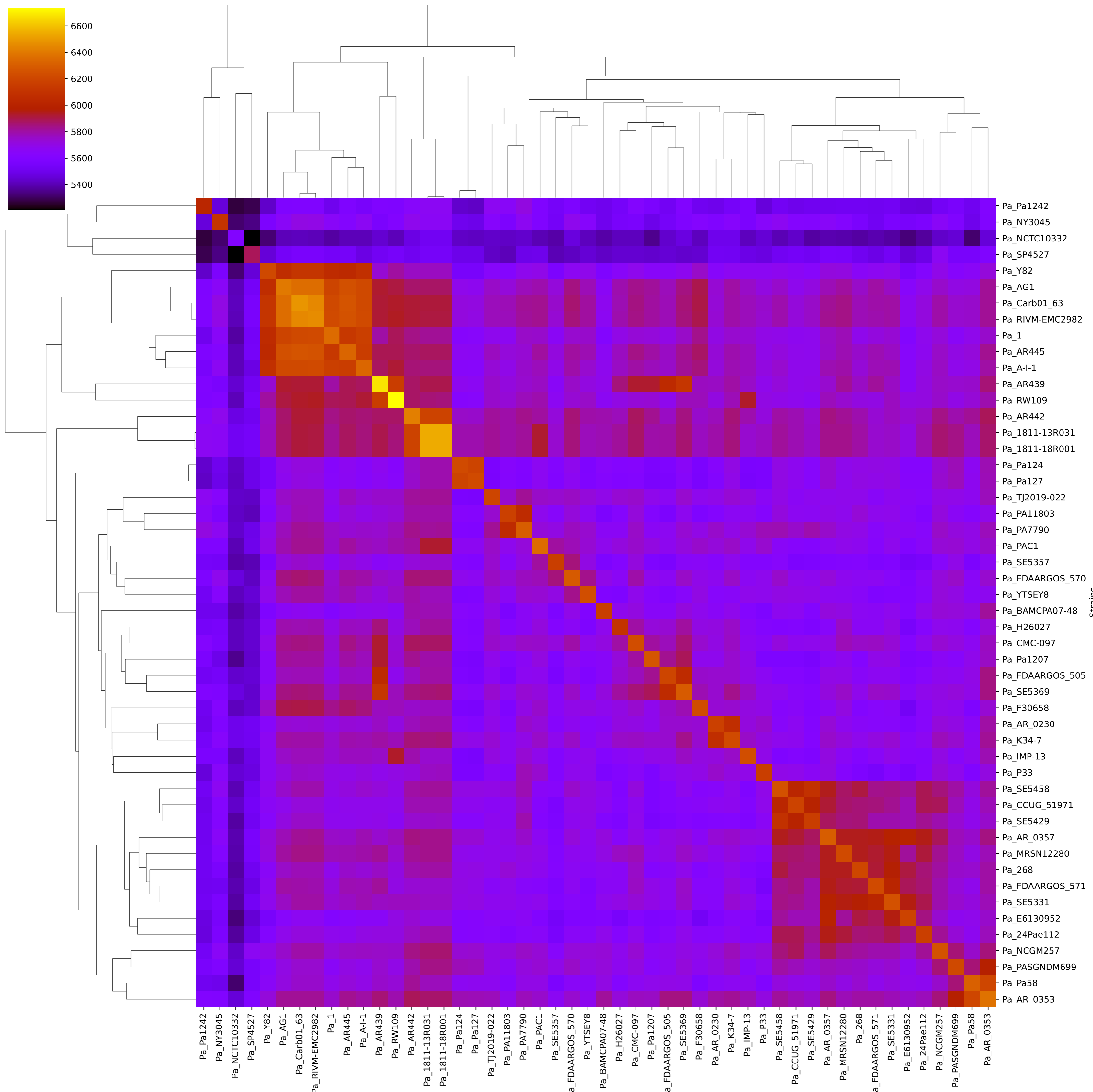

Supplement: Supplementary file 3 [file DataSheet1.pdf]
